# Clinical Genetics Can Solve the Pitfalls of Genome-Wide Investigations: Lesson from Mismapping a Loss-of-Function Variant in *KANSL1*

**DOI:** 10.3390/genes11101177

**Published:** 2020-10-09

**Authors:** Stefania Bigoni, Giuseppe Marangi, Silvia Frangella, Arianna Panfili, Davide Ognibene, Gabriella Maria Squeo, Giuseppe Merla, Marcella Zollino

**Affiliations:** 1 U.O. di Genetica Medica, Dipartimento Materno-Infantile, Azienda Ospedaliero-Universitaria di Ferrara, 44121 Ferrara, Italy; stefania.bigoni@unife.it; 2 Sezione di Medicina Genomica, Dipartimento Scienze della Vita e Sanità Pubblica, Facoltà di Medicina e Chirurgia, Università Cattolica Sacro Cuore, 00168 Roma, Italy; giuseppe.marangi@unicatt.it (G.M.); silvia.frangella@yahoo.it (S.F.); ari.panfili24@gmail.com (A.P.); 3 Fondazione Policlinico Universitario A. Gemelli IRCCS, Unità di Genetica Medica, 00168 Roma, Italy; 4 U.O. di Genetica Medica, Dipartimento di Scienze Mediche, Università di Ferrara, 44121 Ferrara, Italy; gnbdvd@unife.it; 5 Divisione di Genetica Medica, Fondazione IRCCS Casa Sollievo della Sofferenza, San Giovanni Rotondo, 71013 Foggia, Italy; g.squeo@operapadrepio.it (G.M.S.); g.merla@operapadrepio.it (G.M.)

**Keywords:** *KANSL1*, variant interpretation, clinical evaluation, copy number polymorphisms

## Abstract

Massive parallel sequencing of 70 genes in a girl with a suspicion of chromatinopathy detected the (NM_015443.4:)c.985_986delTT variant in exon 2 of *KANSL1*, which led to a diagnostic consideration of Koolen De Vries syndrome. The same variant was present in the healthy mother, consistent with either incomplete penetrance or variant mismapping. A network of second opinion was implemented among clinical geneticists first, and a diagnosis of Koolen De Vries syndrome was considered unlikely. By MLPA, a duplication spanning exons 1-3 of *KANSL1* was detected in both the mother and the daughter. On cDNA sequencing, biallelic wild type mRNA was observed. We concluded that the variant affects the noncoding duplicated gene region in our family, and we finally classified it as benign. Parallel wide genomic sequencing is increasingly the first genetic investigation in individuals with intellectual disability. The c.985_986delTT variant in *KANSL1* was described both in individuals with typical KdVS and in a limited number of healthy subjects. This report highlights the role of clinical genetics to correctly classify variants and to define proper clinical and diagnostic correlations.

## 1. Introduction

The *KANSL1* haploinsufficiency syndrome, also referred to as Koolen De Vries syndrome (KdVS, MIM 610443) is a multisystem disorder characterized by intellectual disability (ID), hypotonia, distinct facial traits, including long face, upslanting palpebral fissures, sparse eyebrows, long and prominent nose with bulbous nasal tip, long philtrum and everted lower lip. Many patients present with friendly and social behavior. Additional component manifestations include epilepsy (50%), short stature (35–40%) and failure to thrive in infancy (35%). Normal head circumference or relative macrocephaly are observed in the majority of patients. KdVS can be caused by either 17q21.31 deletions or truncating variants in the *KANSL1* gene (20%) [1,2,3,4,5,6,7,8]. Of relevance for the precise genetic diagnosis, the first exons of the *KANSL1* gene are included in common duplication polymorphisms. In at least 40% of alleles in the European population, there are one or more extra copies of a genomic region that encompasses either the first three exons (in the so-called H2 inversion haplotypes) or the first four exons (in the H1 haplotypes) of the gene [9,10]. Since the duplicated sequences would code for no functional transcripts, assessing the pathogenicity of loss-of function variants in exons 2–4 of *KANSL1* can be challenging. Notably, several truncating variants in exon 2 of *KANSL1*, including the here described c.985_986delTT, were reported to cause the typical KdVS phenotype [7,8]. Strongly supported by the consistent clinical phenotype, these variants affect the functional copy of the gene.

## 2. Materials and Methods

A 5-year-old girl was referred for genetic evaluation because of clinical manifestations in the spectrum of chromatinopathies, including intellectual disability (ID), autism spectrum disorder, skin abnormalities and distinct facial traits. She is the only child of nonconsanguineous healthy parents. She was born at 38 weeks of gestation by normal delivery. Birth weight was 3170 gr (+2 SD), length was 50 cm (+1 SD) and head circumference was 34 cm (50th–75th centile). Central hypotonia was diagnosed at birth. Motor milestones were delayed; she walked unsupported at age 24 months. She experienced marked language delay; by the age 5 years she was able to speak very short sentences. Neurodevelopmental issues included mild ID, with an IQ of 62, and attention deficit/hyperactivity disorder. She never had seizures, EEG and brain MRI gave normal results. On clinical examination, true microcephaly was noted, with weight of 15 kg (−1.5 SD), height of 112 cm (+1 SD) and head circumference of 46 cm (−3 SD). She presented with mild joint hyperlaxity and mild hirsutism on the back and on the lower limbs and with distinct facial characteristics, including long face with full cheeks, thick eyebrows, bulbous nasal tip, long and prominent philtrum, large and low set ears and micrognathia.

### 2.1. Analyses on Genomic DNA

Genetic analyses were performed for diagnostic purposes and no further authorization was required from the Ethical Committee. Informed consents for genetic testing and the publication of significant results were obtained.

The patient underwent firstly array-CGH (comparative genomic hybridization) analysis with the commercial Agilent 2 × 244 kit (following manufacturer’s instructions, using the ADM-2 algorithm for data analysis with Agilent CytoGenomics software) (Agilent Technologies, Santa Clara, CA, USA), with normal results.

A NGS (Next Generation Sequencing) multigene panel to screen 70 genes responsible for chromatinopathies was subsequently performed with a customised HaloPlex Target Enrichment NGS panel (Agilent Technologies, Santa Clara, CA, USA Agilent Technologies) [11]. Potentially pathogenic variants were confirmed with PCR amplification and Sanger sequencing. DNA from parents was analysed to assess inheritance.

The significance of candidate variants was classified according to the American College of Medical Genetics and Genomics criteria [12] using InterVar (http://wintervar.wglab.org/), Varsome (https://varsome.com/), CAVA and PMut prediction (http://mmb.pcb.ub.es/PMut/) tools. Sequence variants were described according to the Human Genome Variation Society nomenclature guidelines (https://varnomen.hgvs.org/).

### 2.2. Analyses on mRNA

cDNA was obtained by reverse transcription of RNA (High-Capacity cDNA Reverse Transcription Kit, ThermoFisher Scientific) from blood samples of both the patient and her mother. We searched for biallelic expression of *KANSL1* by cDNA sequencing (primers and conditions are available upon request).

## 3. Results

By NGS multigene panel testing, the heterozygous variant c.985_986delTT (NP_056258.1:p.Leu329Glufs*22) in exon 2 of *KANSL1* was selected with the most likely pathogenicity. Variant read depth was 35% of the total at that locus. We detected the same variant in a non-mosaic status in the healthy mother from DNA samples obtained from both peripheral blood cells and buccal smears.

The c.985_986delTT variant in *KANSL1* is reported in the dbSNP (www.ncbi.nlm.nih.gov/snp), with the code rs281865473; in the gnomAD database (gnomad.broadinstitute.org) with a minor allele frequency (MAF) of 0.00002637 in the non-Finnish European population (3 out of 113,762 alleles) and with a MAF of 0.000397 in the Ashkenazi Jewish population (4/10,076); and in the ClinVar database (www.ncbi.nlm.nih.gov/clinvar), with the accession number VCV000038930.1 (variation ID: 38930) as a VUS (variant of uncertain significance), as a de novo pathogenic variant in a patient with the typical phenotype of KdVS described by Koolen and colleagues [8].

MLPA (Multiplex Ligation-dependent Probe Amplification) analysis of *KANSL1* allowed for the detection of the common polymorphic duplication spanning the first three exons of *KANSL1* in both the mother and the daughter.

**Figure 1:**
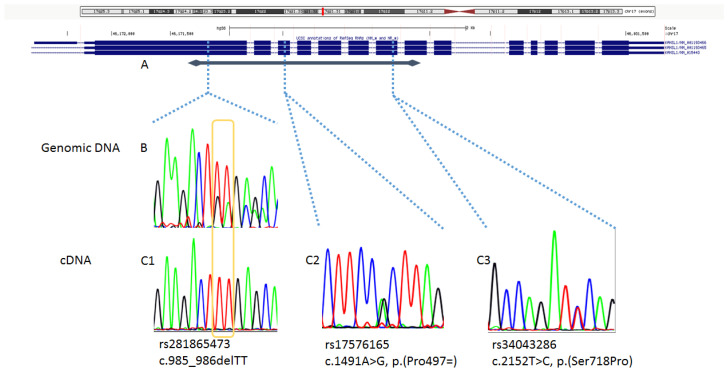
Schematic representation of the sequencing results at both the genomic and cDNA level of *KANSL1*. (**A**) Representation of the genomic region containing *KANSL1*, obtained with Multi-Region visualization (padding of 30 bases) in the UCSC Genome Browser (GRCh38/hg38 Assembly; genome.ucsc.edu). The blue line with diamond shaped ends represents the 1372 bps segment (spanning exons 2-9) of the *KANSL1* cDNA amplified by standard PCR to obtain a unique amplicon. (**B**) Electropherogram of exon 2 sequencing on genomic DNA, showing the c.985_985delTT variant. (**C**) Electropherograms of cDNA sequencing on a large amplicon encompassing exons 2 to 9 of *KANSL1*, showing a wild type exon 2 (**C1**), along with heterozygous c.1491A>G (**C2**, exon 4) and heterozygous c.2152T>C (**C3**, exon 8) variants. These results are consistent with biallelic expression of a functional *KANSL1*.

We searched for biallelic expression of *KANSL1* by cDNA sequencing. The c.985_986delTT variant could not be observed in different amplicons that included the exon 2, likely consistent with nonsense-mediated decay. However, by sequencing wider amplicons, two different common heterozygous variants in exon 4 (rs17576165, c.1491A>G) and in exon 8 (rs34043286, c.2152T>C), respectively, of the gene were detected at the cDNA level, along with wild type exon 2 (Figure 1). On this evidence, nonsense-mediated decay was ruled out, and biallelic expression of wild-type *KANSL1* was assessed. We concluded that the c.985_986delTT (p.Leu329Glufs*22) variant in the present family affects the noncoding duplicated region of the gene, and it was classified as benign.

## 4. Discussion

Congenital neurodevelopmental disorders are highly heterogeneous, both clinically and genetically. Included among neurodevelopmental disorders are a large number of human diseases caused by variants in the different components of the epigenetic machinery, which are referred to as chromatinopathies [13]. Chromatinopathies can undergo phenotypic overlap to each other [14].

For all these reasons, based on the current implementation of genome-wide sequencing, increasingly the first genetic investigation in individuals with ID is next generation sequencing (NGS). However, in the “genotype first” approach, consistency of the genetic diagnosis can be challenging.

NGS analysis of 70 genes was performed in a girl with psychomotor delay and additional clinical manifestations, including skin abnormalities and distinct facial traits, all signs featuring a chromatinopathy, but outside a definite clinical hypothesis. Following the detection of the loss-of-function variant c.985_986delTT (NP_056258.1:p.Leu329Glufs*22) in exon 2 of *KANSL1*, a preliminary diagnosis of Koolen De Vries syndrome was made. The same variant and additional loss-of-function variants in exon 2 of the gene, all de novo, were described in several KdVS patients [7,8].

It is worth noting that, reflecting the complex architecture of the 17q21.31 region containing *KANSL1*, the first three or four exons of the gene are included in common duplication polymorphisms in the European population. Loss-of-function variants affecting these not functional, duplicated copies of *KANSL1* should have no clinical consequences. Accordingly, truncating variants in exon 2 of *KANSL1* are reported in individuals of the general population as well, although not described in association with the polymorphic gene duplication. Nevertheless, very low allele frequency of certain population variants, including the present c.985_986delTT, is described, leading to hypothesize a possible lack of penetrance. Thus, in the evaluation of the ACMG (American College of Medical Genetics) criteria for variant classification, the use of PVS1 (i.e., null variants), PM2 (i.e., absent from control populations), BS1 (i.e., allele frequency greater than expected for disorder) and BS2 (i.e., observed in healthy individuals) criteria should be carefully considered [12]. In fact, variants reported in the first four exons of *KANSL1* in adult healthy controls, and predicted to result in loss-of-function alleles, may actually be located in the polymorphic duplicated region. As a consequence, none of those four evidences could be directly applied.

With respect to our patient, the maternal segregation of the c.985_986delTT (p.Leu329Glufs*22) variant, along with the ascertainment of the polymorphic partial gene duplication where the observed variant could reside, makes the variant most likely benign in nature. However, the most important tool for the definite assessment of its non-pathogenicity was the clinical evaluation of the patient through an implemented second opinion network among clinicians with expertise in this field. Importantly, some clinical manifestations were consistent with a diagnosis of KdVS, in particular the long face with full cheeks, the long and prominent philtrum, the mild ID with marked speech delay, hypotonia and joint hyperlaxity. However, many others were not. Of relevance, our patient presented with microcephaly. Microcephaly is rarely reported in KdVS, in association with chromosome deletions only. On the contrary, normal head circumference or macrocephaly is described in the totality of KdVS patients with *KANSL1* variants [7,8], as consistently as to be included among clinical criteria for the enrolment of non-deleted patients into gene sequencing [7]. On the other hand, we did not observe other typical KdVS features like short stature, friendly behavior and distinct eye and forehead conformation, which in KdvS patients includes short and upslanting palpebral fissures, sparse eyebrows and a pear-shaped nose, thus leading us to exclude the KdVS diagnosis. The regular biallellic *KANSL1* transcription we observed by cDNA sequencing provided the final evidence for this exclusion diagnosis.

Several reasons prompted us to share this case with the scientific community. First, since benign polymorphisms mimicking pathogenic variants in *KANSL1* can be de novo, clinical genetics is proven to play a pivotal role in the precise genetic diagnosis. Secondly, these considerations are mainly addressed to “next gen” geneticists, who are confronted with an increasing amount of high-throughput genome-wide data and will be required to acquire specific expertise in dealing with similar challenges. Finally, collaborations among clinicians and biologists with differentiated expertise in the heterogeneous field of rare diseases should be strongly implemented.

Following the definite assessment of the present *KANSL1* variant as benign, our patient underwent Exome Sequencing with in silico analysis of a large panel of ID genes. A candidate homozygous variant in another gene (GPT2) is under evaluation, but that is a different story.
